# The endocannabinoidome–gut microbiome–brain axis as a novel therapeutic target for autism spectrum disorder

**DOI:** 10.1186/s12929-025-01145-7

**Published:** 2025-07-02

**Authors:** Antonella Campanale, Dario Siniscalco, Vincenzo Di Marzo

**Affiliations:** 1 Department of Psychiatry, McGill University, Montreal, QC H4H 1R2 Canada; 2 Department of Experimental Medicine, Division of Molecular Biology, Biotechnology and Histology, University of Campania, 80138 Naples, Campania Italy; 3 European Biomedical Research Institute of Salerno (EBRIS), 84125 Salerno, Campania Italy; 4 Centre de Recherche de L’Institut Universitaire De Cardiologie Et De Pneumologie de Québec, Département of Médecine, Université Laval, Quebec, QC G1 V 4G5 Canada; 5 Canada Excellence Research Chair On the Microbiome-Endocannabinoidome Axis in Metabolic Health (CERC-MEND), Université Laval, Quebec, QC G1 V 0 V6 Canada; 6 Institut sur la Nutrition et les Aliments Fonctionnels, and Centre NUTRISS, École de Nutrition, Université Laval, Quebec, QC G1 V 0 V6 Canada; 7 Joint International Unit Between the CNR of Italy, Institute of Biomolecular Chemistry, and Université Laval on Chemical and Biomolecular on the Microbiome and its Impact on Metabolic Health and Nutrition (UMI-MicroMeNu), Quebec, QC G1 V 0 V6 Canada

**Keywords:** Autism spectrum disorder, Endocannabinoidome, Gut microbiome, Gut–brain axis, Mental health, Metabolic health, Gastrointestinal health, Therapeutic targets, Biomarkers

## Abstract

**Introduction:**

Autism spectrum disorder (ASD) is characterized by disruption of the gut–brain axis, which leads to behavioral, psychiatric, metabolic and gastrointestinal symptoms. Effective ASD treatments are limited. Research highlights the roles of the endocannabinoidome (eCBome) and gut microbiome (GM), both crucial for brain and gut function. This review summarizes research on therapeutic targets within the eCBome–GM–brain axis for ASD and related comorbidities.

**Discussion:**

Evidence suggests that reduced levels of eCBome mediators, like oleoylethanolamide and anandamide, and altered cannabinoid type 1 and type 2 (CB1 and CB2) receptors activity may contribute to ASD symptoms, making them promising targets. Modulating the eCBome–GM–brain axis with inhibitors of fatty acid amide hydrolase (FAAH), transient receptor potential vanilloid 1, and monoacylglycerol lipase (MAGL) may improve repetitive, stereotypical, and sensory behaviors, and alleviate sociability impairments, depression and anxiety. However, inhibition of FAAH and MAGL may also induce ADHD-like behaviors, which can be reversed by CB1 inverse agonists. Targeting metabotropic glutamate receptor 5 to increase levels of the eCBome mediator 2-arachidonoylglycerol (2-AG) may benefit ASD-related behaviors. eCBome mediators such as 2-AG, 1/2-palmitoylglycerol and palmitoylethanolamide may also help manage ASD- and GI-related symptoms, and systemic inflammation. Other potential therapeutic targets that deserve further investigation are eCBome-related receptors G-protein-coupled receptor 55 and peroxisome proliferator-activated receptors-alpha and -gamma, and the cyclooxygenase-2/prostaglandin E2 pathway, which may address hyperactivity and repetitive behaviors. Additionally, mucin-degrading genera like *Akkermansia* and *Ruminococcus* may improve ASD-related GI symptoms such as hypersensitivity and inflammation. Selective antibiotics against specific *Clostridium* strains may improve irritability and aggression. In ASD with ADHD and OCD, treatments may involve modulating the CB1 and CB2 receptor, and bacterial families like Ruminococcaceae and Lachnospiraceae. Lastly, modulating the abundance of anti-inflammatory genera like *Prevotella* and *Anaeroplasma*, and taxa associated with gut health such as *Roseburia* may also offer therapeutic value.

**Conclusion:**

The eCBome–GM–brain axis is a promising target for ASD treatment, meriting further clinical and preclinical research.

**Supplementary Information:**

The online version contains supplementary material available at 10.1186/s12929-025-01145-7.

## Introduction

Autism Spectrum Disorder (ASD), a neurodevelopmental condition affecting 1 in 36 individuals [1], presents challenges including atypical social communication and interaction, stereotyped language, atypical interests, repetitive and stereotyped behavior [2], and heightened risk for psychiatric and gastrointestinal (GI) comorbidities [3–6]. Struggling to integrate into society, adults with ASD often face unemployment [7], loneliness [8], and financial hardships [9], further exacerbating their already burdened physical and mental wellbeing. Existing behavioral and pharmacological interventions have fallen short in providing evidence of treatment effectiveness [10, 11]. Therefore, investigating novel therapeutic targets for ASD is encouraged. Recently, two emerging sources of chemical signals—the endocannabinoidome (eCBome) and the gut microbiome (GM)—have raised hopes for developing new therapies to address brain dysregulations [12, 13].

The eCBome is an intricate regulatory network that includes the eCB system, with its two major eCBs *N*-arachidonoyl-ethanolamine (anandamide, AEA) and 2-arachidonoyl-glycerol (2-AG), and cannabinoid receptors (CB) of type 1 and 2. The eCBome also includes endogenous and GM-derived eCB-like lipid compounds, a wide array of biosynthetic and inactivating enzymes, receptors, and ion channels, forming a complex web of over 100 mediators, 20 enzymes, and 20 receptors known [14]. The eCBome is involved in social behavior, mood, synaptic plasticity, pain, sleep, GM bacterial composition, gut barrier permeability and gut immunity [15–21], all of which are altered in ASD and can be targeted therapeutically [22].

The GM is the community of microorganisms that resides in the digestive tracts, and is involved in various processes, including: (1) digestion, absorption, detoxification and vitamin synthesis, as well as production of short-chain fatty acids (SCFAs) and other metabolites that are essential for maintaining gut and brain health and providing energy; (2) immune system regulation, inflammation, gut and brain immunity and respective barrier permeability; (3) metabolic functions—the GM influences the host energy balance and plays a role in obesity and metabolic syndrome; and (4) the gut–brain axis and related behavioural, affective and cognitive functions. The bidirectional communication between the GM and the brain involves neural, endocrine, and immune pathways, and has been linked to various neuro-psychiatric conditions, including ASD and its comorbidities [23, 24].

A bidirectional interaction between the eCBome and the GM has emerged recently [25–27], and the effect of this interaction on the brain are altered in states such as ASD. For example, altered eCBome in ASD [22, 28, 29] may reflect changes in GM composition (dysbiosis, a pathological shift of GM composition) [30–32] and vice-versa. Dysbiosis contributes to disruptions in gut immunity, gut barrier integrity, and gut, systemic, and neuroinflammation [33–39], exacerbating GI, systemic, and brain-related symptoms. Certain bacteria and their metabolites can also directly cross the blood–brain barrier, exerting neurotoxic effects [40] and contributing to ASD-like behaviors [41–44]. Some GM strains have been reported to influence the eCB system and exert anti-inflammatory effects [45]. Additionally, some eCB system modulators have shown promising results in alleviating or reversing ASD symptoms in animals [46–53]. While there are several studies on the role of the eCB system in ASD, little is known about the eCBome. Due to its roles, targeting eCBome receptors in ASD may have therapeutic potential at both the gut and brain level [54–58].

The present article examines the eCBome–GM–brain axis as an emerging therapeutic target in ASD.

## Animal models to study the gut–brain axis

Animal models have been critical in investigating the gut–brain axis and its role in neurological conditions, including ASD. Germ-free (GF) mice are among the most commonly used models, as they are raised in a sterile environment devoid of any microorganisms. This allows researchers to observe the impact of the absence of a microbiome on brain function and behavior [59]. These mice can then be colonized with a single or multiple microbial communities to investigate how specific microbes influence the brain, or subjected to a fecal matter transplant (FMT), where feces from one individual or mouse are transplanted into a GF mouse, to examine the role of the gut microbiome specifically.

Conventionalized ex-GF mice, which are initially GF and then colonized with specific microbiota, help researchers explore the impact of particular bacterial communities on brain development and behavior. Monocolonization experiments, where a single bacterial species is introduced into GF mice, help pinpoint the effects of individual microbes on brain function. Also antibiotic treatments can be used to selectively deplete or alter the gut microbiota, allowing studies on how changes in microbial composition affect neurological outcomes [60].

Additionally, more complex approaches like using cocktails of probiotics, which consist of multiple beneficial bacteria, help understand how a diverse microbiome influences the brain [60]. In some studies, a pathogen and a protective strain are introduced to study the interaction between harmful and beneficial microbes and their combined effects on brain function. Vagotomy (severing the vagus nerve) [61] and chemical sympathectomy (disrupting sympathetic nervous system activity through chemical means) [62, 63] can also be employed as a model to block specific neural pathways and explore how gut-derived signals impact brain activity.

Other models also provide valuable insights into the gut–brain axis. Humanized mouse models, where animals are engineered to express human microbiota or genes [64], allow for the study of human-specific microbial influences on the brain [65]. An FMT can reveal how the gut microbiota from different individuals (e.g., ASD) and mouse models (i.e. mice harbouring risk gene mutations for ASD) influences brain function [66]. Dietary interventions are another important tool—by altering the animals’ diet, researchers can investigate how different nutritional components (e.g., high-fat, fiber-rich, “Cafeteria diet”) affect the gut microbiome and, in turn, brain function and behavior [67].

Moreover, neuroinflammation models (e.g., LPS injections) [68] allow for the study of how inflammation in the gut may influence brain function, particularly in the context of ASD. Chronic stress models, such as repeated social stress or maternal separation [69], help examine the role of stress-induced changes in the microbiome on brain activity and behavior [70]. Knockout and transgenic mouse models (e.g., Shank3, Fmr1 knockout mice; addressed in “Genetic models of ASD” section) enable the exploration of gene-environment interactions by investigating how specific genetic mutations associated with neurodevelopmental disorders affect gut–brain signaling (see “Genetic models of ASD” section).

Finally, chronic illness models such as those induced by dextran sodium sulfate (DSS) [71] or trinitrobenzenesulfonic acid (TNBS) [72], which mimic conditions like IBD, can provide insights into how gut diseases or inflammation influence brain development and behavior. Social interaction models, including social defeat or social preference tests, also help examine how microbiome alterations affect social behaviors [73, 74]—an area of particular interest in ASD [75].

These models offer valuable insights into the interactions between the gut microbiome, the brain, and behaviors associated with neurodevelopmental disorders like ASD.

### Use of germ-free mice to assess the impact of specific bacterial species on the gut–brain axis

A number of studies have explored the effect of single-strain colonization in GF mice. While many of these studies focus on the impact of specific bacteria on colonization dynamics throughout the gut and gastrointestinal and systemic inflammation, only a few have investigated their direct effects on the gut–brain axis, influencing behavior and mental health. Philip et al. [76] investigated the effect of *E. coli* strains (JM83, HA107) and *Salmonella enterica serovar Typhimurium* in GF mice. Their study demonstrated that neural plasticity changes rapidly after initial gut microbial colonization and that this process involves innate immune signaling to the brain. The migration of dendritic cells plays a key role in this communication. Nishino et al. [77] observed the colonization effect of strains like *Brauia coccoides* and *Bifidobacterium infantis* on behavior, such as reduced anxiety and locomotor activity. This research supports the notion that gut microbiota can modulate neurotransmitter levels and neurodevelopment, which in turn affect behavior. Liu et al. [78] administered *Lactobacillus plantarum* PS128 in GF mice. The findings demonstrated that live PS128 is safe for chronic ingestion through the gut brain axis and can serve as an anxiolytic agent to regulate the motor functions and mood of the host via modulation of neurotransmitters such as dopamine and serotonin. Parker et al. [79] demonstrated that *Candida albicans* can translocate from the gut to the brain, where it can trigger an inflammatory response. Indeed, in their study, the yeast was often found in close proximity of activated microglial cells. This research supports the idea that disruptions in the gut not only of bacteriome but also of the mycobiome and the intestinal barrier may be important contributors to neurological diseases.

Many other studies have addressed the effect of specific single-strain bacteria on intestinal motility [80], enteric immune activity and systemic inflammation [81–100]. The explored outcomes on immune function could therefore putatively be an indirect index of their potential impact on the gut–brain axis.

## ASD symptoms and etiology

Autism Spectrum Disorder is a neurodevelopmental condition whose core symptoms include atypical social communication and interaction, stereotyped language, repetitive and stereotyped behavior, and atypical interests [101]. Individuals with ASD have up to 78% increased risk of psychiatric comorbidities compared to neurotypical individuals [102]. These include anxiety [103], depressive syndrome [104], obsessive compulsive disorder (OCD) [105], attention deficit hyperactivity disorder (ADHD) [106], as well as atypical behaviors such as sociability impairment [107], sensory behavior [108], hyperarousal [109], irritability [110], tantrum and/or self-injury behavior [111]. Up to 70% of individuals with ASD also suffer from GI [112] and metabolism-related disorders [113] such as gut dysbiosis, digestive enzyme deficits [114] and metabolic syndrome [113, 115].

An epidemiological study from 2022 reports significant variations in ASD prevalence across continents. The highest prevalence is observed in Africa (2963/10,000), followed by Oceania (258/10,000) and the Americas (129/10,000), while lower rates are reported in Asia (34/10,000) and Europe (116/10,000) [116]. These discrepancies may arise from genetic, environmental, and diagnostic differences but also from dietary patterns that shape gut microbiota composition [117].

In Africa, food insecurity and poor maternal nutrition during pregnancy can impair fetal brain development, increasing ASD risk [118]. Additionally, prenatal and early-life exposure to toxins, pollutants, and infections may further contribute to ASD pathophysiology [119, 120]. Diet plays a crucial role in shaping gut microbiota, which in turn influences neurodevelopment. Western diets, rich in fats, sugars, and processed foods, are associated with gut microbiota dysbiosis, increased intestinal permeability, and systemic inflammation—factors implicated in ASD [121, 122]. In contrast, Mediterranean and traditional Asian diets, characterized by high fiber intake and fermented foods, promote microbial diversity and the production of short-chain fatty acids (SCFAs), which exert metabolic [123] and neuroprotective effects [124–126]. Notably, Western dietary patterns have been linked to cognitive decline, whereas Mediterranean diets have demonstrated cognitive benefits [127]. Thus, epidemiological evidence indirectly supports the link between GM composition and ASD symptom severity and prevalence.

### Animal models employed to investigate ASD symptoms and etiology

ASD is highly heterogeneous not only in its phenotypic presentation but also in its etiology. The disorder is often classified into different sub-populations based on genetic and epigenetic modifications [128]. Preclinical models of ASD replicate certain phenotypes through genetic, epigenetic, or combined factors, providing controlled environments to study the disorder’s complexities and test hypotheses. These models can be divided into two main categories: genetic models and environmental models [129].

#### Genetic models of ASD

The genetic architecture of autism is complex and lacks a single causative gene, but clear genetic bases are identifiable in about 20–25% of cases [130]. ASD often co-occurs with rare Mendelian neurodevelopmental disorders such as fragile X syndrome (FXS) [131, 132], Rett syndrome (RTT) [133, 134], neurofibromatosis type 1 (NF1) [135], and tuberous sclerosis complex (TSC) [136] as well as copy number variations like those in Phelan–McDermid syndrome, which involves mutations in SH3 and multiple ankyrin repeat domains 3 (SHANK3) [137].

Fragile X syndrome is caused by an expanded CGG trinucleotide repeat in the fragile X messenger ribonucleoprotein 1 (FMR1) gene, leading to disrupted synaptic function [131] and ASD-like behavioral phenotypes such as intellectual disability, hyperarousal, and impulsivity [132]. Mutations in the methyl-CpG binding protein 2 (MECP2) gene cause Rett syndrome, leading to developmental regression, motor and language impairments, stereotypic movements, seizures, and autonomic dysfunction [133, 134]. Autosomal dominant mutations in the neurofibromin 1 (NF1) gene cause tumor formation and neuronal dysfunction, leading to cognitive, language, and behavioral deficits, with high rates of ASD [135]. Mutations in tuberous sclerosis complex 1 (TSC1) and tuberous sclerosis complex 2 (TSC2) genes disrupt neuronal development, resulting in seizures, intellectual disability, and ASD in about 50% of affected individuals [138]. *SHANK3* mutations in Phelan–McDermid syndrome results in severe expressive language delays, hypotonia, and ASD-related behaviors [137].

Rodent models genetically engineered to mimic genetic forms of ASD offer a means to directly study the genetic contributions to ASD, investigating the behavioral phenotypes observed in ASD. It is important to note that not all ASD models exhibit robust construct validity. For example, models like the BTBR and C58/J mouse naturally display idiopathic autism-like behaviors. In such cases, the model validity is assessed based on face validity. Ultimately, no single animal model can entirely capture the complexity of ASD. However, collectively, these models provide a way to approximate it [139].

#### Environmental models of ASD

Despite high heritability, the lack of perfect concordance between monozygotic twins [130] suggests that environmental factors also play a role in the etiopathogenesis of ASD. Key environmental risk factors during the perinatal period include advanced parental age, maternal diabetes, maternal infections, medication use during pregnancy, and toxin exposure, though their associations with ASD vary [140, 141]. Therefore, these environmental models of ASD study epigenetic causes of autism mainly focusing on the maternal immune system activation (MIA) and prenatal exposure to medications and toxins [142]. Exposure to valproic acid (VPA), a widely studied antiepileptic and mood stabilizer, serves as a model of MIA during pregnancy, along with other compounds like thalidomide, misoprostol, and propionic acid [143]. These environmental models are often combined with genetic modifications to explore *gene* × *environment* interactions.

#### Germ-free mice to investigate the gut–brain axis in ASD

GF mice have been instrumental in dissecting causal relationships between gut microbes and ASD-like behaviors. In some studies, these mice have been found to exhibit social deficits, increased anxiety-like traits, and altered brain development, mirroring core ASD phenotypes [59]. Mechanistically, the absence of microbiota in GF mice may affect microglial maturation, HPA axis regulation, neurotransmitter systems, and immune activation, all of which contribute to ASD pathophysiology [144, 145]. When gut microbiota are reintroduced into these mice, researchers can observe the effects of specific bacterial species on ASD-related behaviors. For instance, *Bifidobacterium infantis*, a species commonly used in microbiota studies, has been shown to alleviate anxiety-like behaviors and stress responses in GF mice, indicating its potential role in modulating behavior [77, 144, 145].

FMT studies in GF mice also provide insights into how specific bacteria contribute to ASD-related behaviors. FMT from healthy donors to GF mice has been shown to improve social behaviors, whereas FMT from ASD donors can induce ASD-like behaviors. For example, Xiao et al. [146] demonstrated that microbiota from ASD individuals induced ASD-like behaviors in GF mice, with certain bacterial species likely playing a key role in these effects. These findings suggest that specific bacteria in the gut microbiota can influence ASD-like behaviors, providing a preliminary picture of how individual species contribute to neurodevelopment and behavior in preclinical models.

### The influence of gut microbiota on preclinical models of ASD

Gut microbiota plays a critical role in shaping preclinical models used to study ASD, for example by altering intestinal barrier integrity [147–151], immune responses, neuroinflammation [152–154], and neural signaling [155]—factors that contribute to ASD-like behaviors. However, despite clear associations between gut dysbiosis and ASD phenotypes [156], findings across ASD models remain inconsistent [157]. Differences in genetic background, environmental conditions and sex may contribute to these discrepancies, affecting the reproducibility and interpretation of microbiota-driven effects in ASD research.

One key mechanism through which microbiota influences ASD models (both genetic [147, 148], environmental [149] and idiopathic ones [150, 151]) is the disruption of gut barrier integrity, leading to systemic inflammation and altered brain function. These models display increased intestinal permeability [99–103], yet some studies report no significant differences, suggesting that microbiota-driven barrier dysfunction may be strain- or condition-dependent [157]. Additionally, gut microbiota composition differs across ASD models, with alterations in α-diversity, β-diversity, and specific taxa linked to ASD-like traits (reviewed in [156]). While some studies report consistent microbial signatures associated with ASD, others find conflicting patterns, even within the same model [156], emphasizing the complexity of microbiota contributions. Sex differences further modulate these findings, influencing microbiota composition and ASD-related behaviors in genetic [158, 159], environmental [160–163], and idiopathic [150] models.

Microbiota-derived metabolites, particularly SCFAs, further shape ASD models. Butyrate, which modulates gut integrity and neuroinflammation [152, 164], is elevated in several environmental [160], genetic [159], and idiopathic [151] models of ASD. However, not all studies report this increase—Rett syndrome models, for instance, show no significant changes in butyrate levels [165]. Interestingly, sodium butyrate administration improves atypical behaviors and cognition in BTBR [33, 166] and VPA models [167], yet its effects on gut microbiota composition in ASD remain unclear, with some studies suggesting modulation of gut microbiota and permeability in environmental model-specific responses [168–170]. Conversely, propionate (PA), another SCFA, consistently induces ASD-like behaviors in rodents, highlighting how microbial-produced SCFAs can either mitigate or worsen ASD traits depending on the model.

Microbiota-targeted interventions, including probiotics and antibiotics, further illustrate the variability in microbiota-immune-brain interactions across ASD models. While *Bacteroides fragilis* restored gut permeability and improved behavioral deficits, including reducing repetitive behaviors and anxiety-like symptoms, in MIA models [149], it did not fully rescue social deficits, highlighting the model-dependent limitations of microbiota-based interventions. Similarly, *Lactobacillus reuteri* improved social and repetitive behaviors in multiple (genetic [157, 158], idiopathic [157], and environmental [157, 171]) ASD models. Antibiotic studies also show interesting results—vancomycin treatment in MIA models improved ASD-like behaviors, sociability and anxiety [172], streptomycin rebalanced gut dysbiosis and memory deficits in another MIA model [173], while neomycin improved social behavior in a genetic model [174].

Co-housing studies demonstrated how gut microbiota can influence ASD-like traits in preclinical maternal environmental models [175] through microbial exchange via coprophagy and grooming [176]. Early life is a critical period for these interactions, which may shape neurodevelopment. For example, Lammert et al. [175] showed that offspring of segmented filamentous bacteria (SFB)-negative dams exhibited ASD-like behaviors only when co-housed with SFB-positive dams, indicating the importance of microbiota exchange in model outcomes. Additionally, Buffington et al. [171] found that co-housing maternal high-fat diet offspring with control mice improved social behaviors and microbiota composition, suggesting a microbiota-driven rescue effect. These findings highlight the potential of co-housing as a method to study the role of microbiota in ASD models, though further research is needed to confirm these effects.

## The eCBome–GM–brain axis as a model to investigate therapeutic targets in ASD and comorbidities

Individuals with ASD suffer from both gut- and brain-related comorbidities. Gastrointestinal symptoms in ASD are thought to be caused by central sensory augmentation, altered modulation of GI epithelial permeability including visceral hypersensitivity, and altered enteric nervous system-mediated motility and secretion [177]. Increased permeability and disruption of gut and blood–brain barrier integrity are observed in individuals with ASD [178, 179]. This permeability results in a higher antigenic load from the GI tract entering circulation and crossing the BBB, inducing a brain immune response [180, 181]. Pro-inflammatory mediators may directly communicate with microglia through the neurovascular network, creating a reactive environment that negatively affects the functioning of neuronal circuits responsible for mood regulation [182].

Immune dysfunction, gut dysbiosis, and the eCBome in ASD are closely intertwined. Gut dysbiosis contributes to immune activation and exacerbates intestinal permeability and inflammation, leading to GI symptoms [179, 183]. The GI system maintains a close connection with the brain through the eCBome, primarily mediated by CB1 receptors and transient receptor potential cation channels subfamily V member 1 (TRPV1) in myenteric and vagal fibers [184, 185], peroxisome proliferator-activated receptor alpha (PPAR-α), and oleoyl-lysophosphatidylinositol receptor 1 (GPR119) in enteroendocrine epithelial cells of the small intestine [186, 187]. These receptors influence myenteric neuron activity, nerve function, and the release of GI and neuroactive neuropeptides. Dysbiosis in ASD affects mental health via the GM production of molecules that influence cognitive or social behaviors, impacting myenteric and vagal nerve activity directly or via the bloodstream, exacerbating ASD and systemic symptom severity [180, 188, 189].

Notably, neurotransmitters like serotonin or gamma-aminobutyric acid (GABA), and eCB-like mediators such as *N*-acyl amides that interact with G-protein-coupled receptors, produced from commensal microorganisms, can modulate host eCBome receptors [190–193]. Additionally, significant changes in GM abundance and composition can alter the expression of eCBome receptors and enzymes, and the concentrations of eCBome mediators in the gut, influencing the eCBome–GM–brain axis [27, 194]. For instance, antibiotic-induced dysbiosis alters the levels of eCBs and their mediator congeners, ultimately leading to decreased neurogenesis and ASD-like behaviors, which can be normalized by treatment with probiotics such as *Lactobacillus casei* [195]. Specific GM taxa influence the eCB system, inducing the expression of CB1 and CB2 receptors, leading to anti-inflammatory and analgesic effects [45, 196]. The administration of various strains of *Lactobacillus* and *Bifidobacterium* (genera underrepresented in ASD) have shown promise in improving ASD symptoms and gut integrity in both preclinical and clinical studies [149, 171, 196, 197]. Indeed, several clinical trials administering probiotics containing *Lactobacillus* and *Bifidobacterium* strains (NCT04939974, NCT06448767, NCT04942522, NCT03514784, NCT02903030, NCT03369431, NCT02708901, NCT04293783, NCT06126185, NCT06650644, NCT05307744, NCT03982290) are ongoing or were recently completed. Others have administered *Lactobacilli* and *Bifidobacteria* in combination with prebiotics (NCT06126185, NCT04944901, NCT05151601, NCT02086110, NCT04639141). Fecal microbiota transplant-based therapies in children and adults with ASD are also under investigation (NCT06419530, NCT03426826, NCT04246398, NCT03408886, NCT04630847, NCT06030752, NCT04948814, NCT06290258, NCT02504554, NCT06503978, NCT04182633, NCT03829878).

### The eCBome–GM–brain axis in ASD, anxiety, sociability and behavioral impairment

Gut microbial diversity is significantly disrupted in ASD, with a reduced ratio of Bacteroidetes to Firmicutes and an increase in Proteobacteria [198, 199]. High levels of Proteobacteria in ASD, including opportunistic LPS-producing pathogens, may lead to systemic inflammation and impair antioxidant mechanisms [34, 35]. These changes in turn can contribute to altered social behavior and anxio-depressive tendencies [200], comorbidities often manifested in ASD. Given the involvement of inflammation in ASD pathophysiology, increasing endocannabinoid (eCB) tone has been proposed as a potential therapeutic strategy [16, 201].

Individuals with ASD exhibit reduced serum levels of the eCBs anandamide (AEA) and oleoylethanolamide (OEA) [29]. Notably, OEA and AEA increase oxytocin signaling [202–204]. Oxytocin administration was reported to improve several ASD symptoms and behaviors, such as enhancing social cognition [205, 206] and behavior [207], emotional recognition of facial expressions [208], and reducing anxiety [209–213]. AEA by increasing oxytocin led to social reward and improved social impairment in ASD mouse models [53, 204]. Therefore, interventions that restore AEA levels—such as CB1 receptor activation or inhibition of fatty acid amide hydrolase (FAAH) (the enzyme responsible for degrading AEA [214–216])—may help mitigate these symptoms [53, 204]. In preclinical models, increasing eCB signaling via CB1 activation has been shown not only to enhance social reward [53], but also to reduce aggression [217], and stress-induced impulsivity [218, 219]. These ASD-related symptoms will be discussed in detail in the next section.

However, the effects of CB1 modulation are highly context-dependent, as CB1 receptors regulate both GABAergic (inhibitory) and glutamatergic (excitatory) neurotransmission [220]. In some cases, CB1 antagonists (which reduce CB1 activity) have been reported to exert either anxiolytic or anxiogenic effects, depending on which neurotransmitter system they predominantly affect. Specifically, CB1 blockade reduces anxiety when targeting GABAergic pathways but increases anxiety when interfering with glutamatergic ones [220] (Table 1). These findings suggest that the effect of CB1 modulation in ASD may depend on the specific neurochemical transmission and alterations present in different ASD subtypes.

**Table 1:** The degenerative and therapeutic role of the eCBome in ASD

eCBome role	Key findings	Model	References
Degenerative	Reduced eCB levels, increased eCBome enzyme degradation, and upregulated cannabinoid receptors (CBRs) contribute to ASD-like behaviors such as hyperactivity, repetitive actions, and social dysfunction	Children with ASD and in the VPA-induced animal model	[47]
	Reduced levels of anandamide (AEA) promote anxiety	Male Sprague–Dawley rats	[214]
	CB1 antagonist rimonabant caused depressive-like effects	Male Wistar rats	[216]
	CB2 inhibition increases aggression, preferentially in female mice	CB2 receptor KO mice [286] Sprague–Dawley rats [287]	[286, 287]
	CB1 receptor KO mice exhibited increased context dependent-anxiety-like behavior, sociability impairment and aggression	CB1 receptor KO mice	[317]
	Excessive AEA levels can activate TRPV1 receptors and exacerbate anxiety	TRPV1 receptor KO C57BL/6 J mice	[197]
	Chronic administration of CB1 receptor agonists during adolescence but not during adulthood impairs emotional behaviour and monoaminergic neurotransmission	Sprague–Dawley rats	[243]
	Disruptions in the functional activity of mGlu5Rs may underpin synaptic dysfunction and behavioral deficits in ASD	Shank 3 knockout mice	[249]
	PPARγ downregulation enhances fear, emotional response to acute stress and exacerbates anxiety	PPARγ^NestinCre^ KO mice [253] C57BL/6 J mice [255]	[253, 255]
	PPARα downregulation enhances fear, emotional response to acute stress and exacerbates anxiety	PPARα KO mice [254] C57BL/6 J mice [255]	[254, 255]
	Abnormal COX2/PGE2 signaling in COX2 deficient mice leads to autism-related behaviors, with males showing hyperactivity, anxiety, and repetitive behaviors, and females exhibiting social impairments	COX2-deficient mice	[274, 275]
	COX2-deficiency leads to autism-related behaviors, such as hyperactivity and atypical communication	Children with ASD	[278]
	Misuse of misoprostol (a PGE2 analogue) during early pregnancy is linked to a higher incidence of neurodevelopmental disorders, including Moebius syndrome and ASD	Children with ASD	[279–281]
	Altered AEA degradation, due to decreased FAAH activity in lymphocytes is associated with attention-deficit/hyperactivity disorder (ADHD)	Adolescences with ADHD	[295]
	CB1 receptor blockade increases anxiety when interfering with glutamatergic function	CD1 mice and Wistar rats	[220]
	Striatal CB1 receptors on GABA terminals are insensitive in ADHD and do not respond to their agonists	DAT–CI mice	[296]
	FAAH and MAGL inhibition causes ADHD-like behaviors	SHR rats	[298]
	mGluR5 activation induces long-term depression at presynaptic sites, reducing synaptic transmission and contributing to neuronal dysfunction in the non-activated pathway	Visual cortical slices from mice	[306]
	DAGLα and NAPE-PLD enzymes, CB1, CB2 and oxytocin receptors are downregulated in OCD	DAT heterozygous rats with compulsive behaviors and individuals with OCD	[309]
Therapeutic	Increasing anandamide (AEA) signaling, through anandamide-deactivating enzyme FAAH inhibition ameliorates social disabilities	Male Wistar rats [16] Unspecified male mice [53] BTBR and fmr1^−/−^ mice [53] VPA-mice [52, 237, 238]	[16, 52, 53, 237, 238]
	AEA-mediated signaling at CB1 receptors, driven by oxytocin, controls social reward and improves social impairments	C57Bl6 J male mice [204] BTBR and fmr1^−/−^ mice [53]	[53, 204]
	CB1 receptor blockade reduces anxiety when targeting GABAergic pathways	CD1 mice and Wistar rats	[220]
	Low doses of the CB1 receptor antagonist rimonabant and the neutral antagonist NESS0327 normalize memory deficits and cognitive impairments	Fmr1 KO mice	[49]
	Increasing eCB signaling via CB1 activation reduces aggression, and stress-induced impulsivity	Male C57BL/6 JNarl mice [217] Male CB1 KO [283]	[217, 283]
	CBD rescues cognitive deficits by enhancing short-term memory through GPR55 receptor activation in the hippocampus and modulating anandamide metabolism	Fmr1-Δexon 8 rats	[318]
	Beta-caryophyllene-induced CB2R activity mediates antidepressant, and memory-enhancing effects	Wistar rats	[232]
	CB2 receptor activity leads to the upregulation of PGC-1α and PPAR-γ activity, exerting anxiolytic and anti-inflammatory effects	Wistar rats	[232]
	CB1 receptor agonists exert antidepressant effects	Male Long-Evans rats [240] Male BALB/c mice [241] Male Sprague–Dawley rats [242]	[240–242]
	FAAH inhibition improves aversion memory and relieves anxiety behavior	Wistar Kyoto rats [236] VPA-mice [52]	[52, 236]
	Activation of the endocannabinoid system via CB1 receptor agonists, FAAH inhibitors, or eCB uptake inhibitors exerts antidepressant-like effects and enhances the efficacy of traditional antidepressants	Male Wistar rats	[216]
	2­AG signaling enhanced by a monoacylglycerol lipase (MAGL) inhibitor reduces aggressiveness and increases victimization	Male CD1 mice	[284]
	CBD decreases social isolation-induced aggressive behaviors potentially by increasing the activation of 5-HT1 A and CB1 receptors	Swiss mice	[285]
	Acute administration of CB2 agonism significantly reduced isolation-induced aggressivity	OF1 mice	[286]
	2­AG signaling enhanced by a MAGL inhibitor reduces OCD-like behavior	Sprague–Dawley rats	[287]
	Increasing AEA signaling, through FAAH inhibition ameliorates communication deficits	VPA-mice	[52]
	Increasing AEA signaling, through FAAH inhibition ameliorates repetitive behavior	VPA-mice	[238]
	Dual FAAH/TRPV1 show more anxiolytic effects than selective FAAH inhibitors alone	TRPV1-KO C57BL/6 J mice	[197]
	Selective inhibitors of MAGL improve repetitive and stereotypical behaviors, hyperactivity, and anxiety-like behavior	VPA mice [47] Fmr1^−/−^ mice on a C57BL/6 J [246] Fmr1 KO mice [247]	[47, 246, 247]
	Increasing 2-AG levels through a selective inhibitor of MAGL improves sociability, social preference, and cognitive functioning	VPA mice	[47]
	Selective inhibitors of MAGL improve sensory behavior	Fmr1 KO mice	[247]
	CB1 receptor antagonim improved memory impairments	Fmr1 KO mice	[49]
	Palmitoylethanolamide (PEA) supplementation improves repetitive and stereotyped behaviors and sociability	BTBR T + tf/J mice [54] VPA mice [250] BTBR T + tf/J mice [54]	[54, 250]
	PEA supplementation enhances language skills, and improves sensory sensitivity and sociability	Children with autism	[251]
	PEA reduces repetitive behavior and increases sociability while mitigating GI inflammatory disorders and intestinal permeability	BTBR T + tf/J mice	[54]
	Amygdala PPARγ activity reverses emotional response to acute stress and anxiety	PPARγ^NestinCre^ KO mice	[253]
	GPR55 receptor agonists exert anxiolytic-like effects in the medial orbital cortex of mice with acute stress	Male C57BL mice	[256]
	PPAR-α antagonists significantly attenuate social impairment, repetitive behavior, hyperactivity, anxiety, and low exploratory activity in environmental models of ASD	VPA mice [257] PPA-treated rats [258]	[257, 258]
	Increased 2-AG levels normalize sensory behavior and improve anxiety-like and hyperactivity behaviors	Fmr1 KO mice	[247]
	A CB1 receptor inverse agonist (SLV-319) causes a reduction of ADHD-like behaviors such as hyperactivity and anxiety	SHR rats	[298]
	Endocannabinoids and CB1 receptors (activation) upregulate heterosynaptic long-term depression, contributing to synaptic plasticity. BDNF prevents the suppressive effects of eCBs, maintaining transmitter release at presynaptic sites	Visual cortical slices from mice	[306]
	BDNF triggers the release of eCB, which act on CB1 receptors at presynaptic GABA terminals to regulate inhibitory synaptic transmission	Neocortical slices from mice	[307]
	eCB and CB1 receptors activation facilitate long-term potentiation at pyramidal neurons, contributing to synaptic plasticity. BDNF enhances this process, promoting sensory information processing	Thalamocortical slices from rats	[308]

Summary of key findings on the degenerative and therapeutic role of the endocannabinoidome (eCBome) in various behavioral and neuropsychiatric conditions occurring in autism spectrum disorder (ASD). The table outlines the observed eCBome alterations in different animal models and human samples, highlighting behavioral, biochemical, and functional changes. The table also provides information about the findings, model, and references

*eCB* endocannabinoid, *CBR* cannabinoid receptor, *AEA* anandamide, *CB1* Cannabinoid receptor 1, *CB2* cannabinoid receptor 2, *TRPV1* transient receptor potential vanilloid 1, *mGlu5R* metabotropic glutamate receptor 5, *PPARγ* peroxisome proliferator-activated receptor gamma, *PPARα* peroxisome proliferator-activated receptor alpha, *COX2* cyclooxygenase 2, *PGE2* prostaglandin E2, *FAAH* fatty acid amide hydrolase, *MAGL* monoacylglycerol lipase, *OCD* obsessive–compulsive disorder, *2-AG* 2-arachidonoylglycerol, *GABA* gamma-aminobutyric acid, *GPR55* G-protein-coupled receptor 55, *BDNF* brain-derived neurotrophic factor, *SHR* spontaneously hypertensive rat, *DAT–CI* dopamine transporter–cocaine interaction, *KO* knockout, *VPA* valproic acid, *BTBR* brain-targeted receptor, *Fmr1* fragile X mental retardation 1, *OF1* outbred field mice, *SLV-319* selective ligand for variant 319, *PEA* palmitoylethanolamide

Interestingly, gut microbiota may further contribute to ASD-related disruptions in CB1 signaling. In clinical studies, lower oxytocin serum levels in ASD correlate with increased relative abundance of *Clostridia* genera [221]. These bacteria, along with *Desulfovibrio* (also elevated in ASD) [198, 222], are propionate-producing bacteria [223]. Excessive levels of propionate induce dysbiosis, neurotoxicity, and ASD-like behaviors [224–226], potentially by altering neurotransmitters such as dopamine, GABA, and serotonin [227–230]. Intriguingly, blocking CB1 receptors in the gut increases propionate-producing bacteria, though it also elicited beneficial effects on metabolic syndrome [231].

Beyond CB1, CB2 receptors have emerged as a promising therapeutic target for treating anxiety, depression, and memory deficits in ASD, along with the associated metabolic comorbidities. Specifically, beta-caryophyllene, an agonist of CB2 and PPAR-γ receptors, improved not only metabolic parameters but also psychiatric and behavioral symptoms by exerting anti-inflammatory effects in the PFC of rats with neuroinflammation and metabolic syndrome [232].

Anxiety and depression often involve an overexpression of FAAH in the amygdala and hippocampus [214–216]. Indeed, in animal models of ASD, FAAH inhibitor improved aversion memory and relieved anxiety behavior [233] and depression [216, 234–236], reversed social impairment [52, 53, 237, 238], communication deficits, stereotyped behavior [52], and repetitive and atypical emotion-related behaviors [238]. However, excessive AEA levels can activate TRPV1 receptors, which may paradoxically exacerbate anxiety. This explains why dual FAAH/TRPV1 blockers have shown more consistent anxiolytic effects than selective FAAH inhibitors alone [239].

**Fig. 1:**
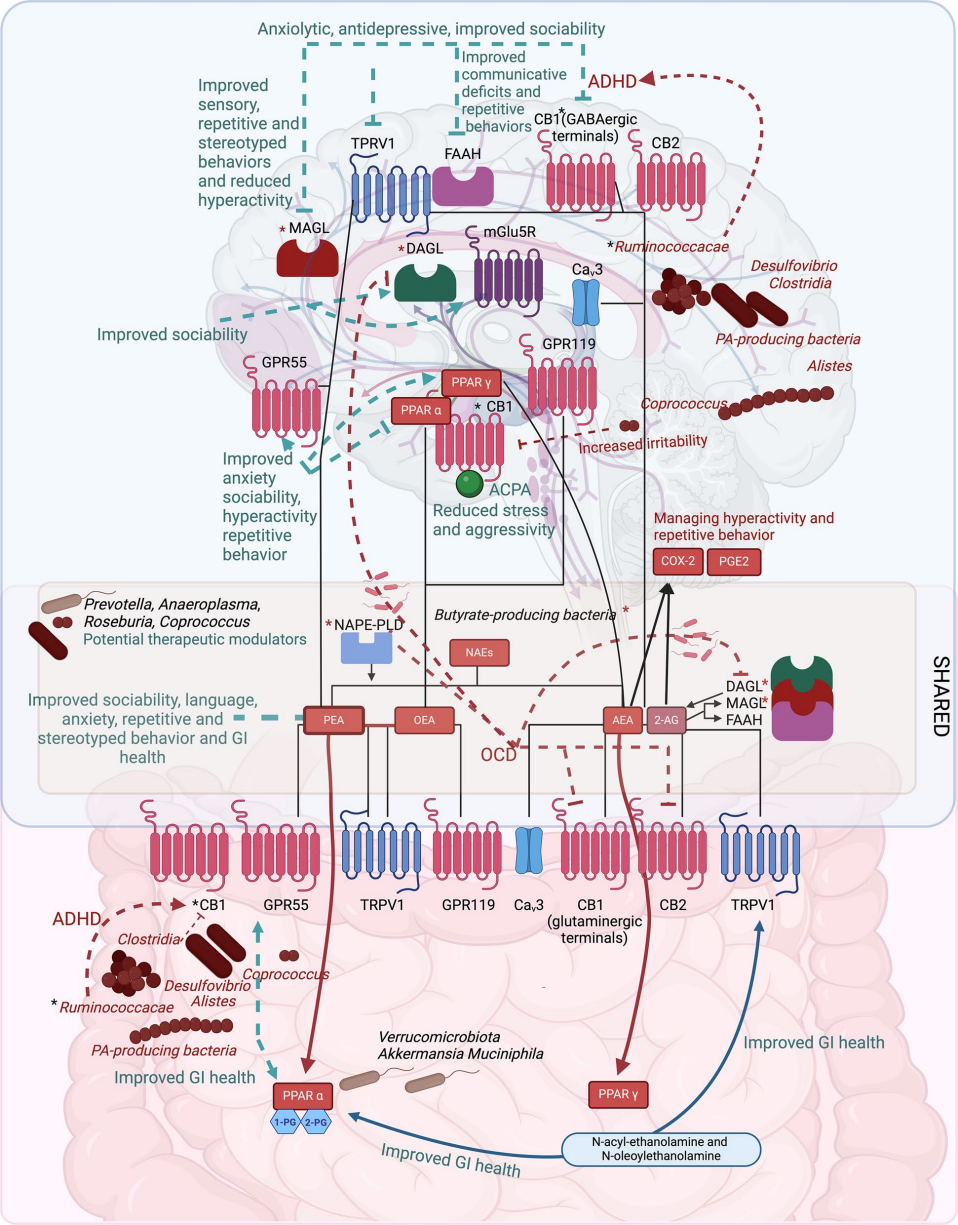
The endocannabinoidome (eCBome)–gut microbiome–brain axis in autism spectrum disorder and related comorbidities. The brain is represented in the upper part, while the gut is represented in the lower part. In both organs, the eCBome receptors, ion channels and enzymes are present. The eCBome mediators most investigated are shown. Black lines indicate their binding activity with the respective target receptors, ion channels and enzymes. Gut microbial taxa involved in gut–brain axis communication are graphically designed. Green arrows indicate a therapeutic modulation; red arrows indicate a pathological modulation. Lines terminating with a dash indicate an activity inhibition, while those terminating with an arrow indicate an activation. The asterisk indicates a modulation whose effect is not clear. Propionic acid (PA), Obsessive compulsive disorder (OCD), Attention deficit hyperactivity disorder (ADHD), Serotonin (5-HT), Dopamine receptors D1-3 (D1-3), metabotropic glutamate receptor 5 (mGlu5R), 1-Palmitoyl-glycerol (1-PG), 2-Palmitoyl-glycerol (2-PG), Peroxisome proliferator-activated receptor-alpha (PPAR-α), Peroxisome proliferator-activated receptor-gamma (PPAR-γ), *N*-acyl phosphatidylethanolamine phospholipase D (NAPE-PLD), Fatty acid amide hydrolase (FAAH), Diacylglycerol-lipase (DAGL), Monoacylglycerol lipase (MAGL), *N*-acylethanolamines (NAEs), Palmitoylethanolamide (PEA), Oleoylethanolamide (OEA), Anandamide (AEA), 2-Arachidonoylglycerol (2-AG), Arachidonylcyclopropylamide (ACPA), Transient receptor potential vanilloid 1 (TRPV 1), Gamma-aminobutyric acid (GABA), Cannabinoid type 1 receptor (CB1), Cannabinoid type 2 receptor (CB2), G protein-coupled receptor 55 (GPR55), G protein-coupled receptor 119 (GPR119). Created with BioRender.com

Thus, we speculate that the abundance of propionate-producing bacteria and the downregulation of CB1 receptors in the gut, along with decreased concentrations of OEA and AEA, may contribute to ASD symptoms and pathophysiology. Targeting this axis and modulating it with eCBome enzyme/receptor (such as FAAH/TRPV1) blockers may have therapeutic potential in ASD-related anxiety and depressive tendencies as well as behavioral and sociability impairment (Fig. 1).

Preclinical studies further suggest that CB1 receptor agonists exert antidepressant effects [240–242], though findings in adolescent rats remain controversial [243]. CB2 receptor agonists such as cannabidiol (CBD) may also hold therapeutic value for ASD (see Supplementary Table 1). Overall, the differential roles of CB1 in ASD can be understood in terms of regional and neurotransmitter-specific mechanisms. CB1 activation may be beneficial for social behavior, aggression, and reward processing, whereas CB1 inhibition could be useful for hyperactivity-related symptoms (e.g., ADHD-like behaviors). Meanwhile, CB2 activation, FAAH inhibition, and dual FAAH/TRPV1 blockade may expand the therapeutic potential of eCB-based interventions in ASD.

In addition to OEA and AEA, palmitoylethanolamide (PEA) and 2-arachidonoylglycerol (2-AG) are also found at reduced levels in serum [29, 244], while CB2 receptor expression is increased in peripheral blood mononuclear cells in individuals with ASD [245]. Selective inhibitors of monoacylglycerol lipase (MAGL), an enzyme that breaks down 2-AG, improved repetitive and stereotypical behaviours, hyperactivity, anxiety-like behavior [246, 247], sociability, social preference, cognitive functioning [47], and sensory behavior [247].

In the *Fmr1* model of ASD, which recapitulates fragile X syndrome (FXS), a failure of diacylglycerol Lipase α (DAGL-α), the main enzyme responsible for synthesizing 2-AG, was associated with metabotropic glutamate 5 receptor (mGlu5R) deficits [248]. Additionally, disruptions in the functional activity of mGlu5Rs was observed in the *Shank 3* knockout model of ASD [249]. We speculate that increasing mGlu5R activity may enhance the activity of DAGL, thereby raising 2-AG levels, and exerting beneficial effects on ASD symptoms.

Studies on PEA demonstrate its dual effects on both the gut and brain: its supplementation improved repetitive and stereotyped behaviors and sociability in ASD models [54, 250], enhanced language skills, and improved sensory sensitivity and sociability in children with autism [251], while mitigating GI inflammatory disorders and intestinal permeability [54]. These effects are possibly elicited through activity at its proposed targets, i.e. G protein-coupled receptor 55 (GPR55) [252], PPAR-α [54], and indirectly via enhancement of eCB levels or activity [252]. Two clinical trials administering PEA are ongoing (NCT06187090) and, of which one (NCT05182697) administers PEA in combination with cannabidiol to obtain an “entourage effect”. Brain PPAR-γ and PPAR-α are implicated in emotional stress and anxiety in preclinical models [253–255]. GPR55, which is activated by both eCB and non-eCB ligands, plays a role in anxiety regulation [256], suggesting a broader involvement of the eCBome in anxiety modulation. Interestingly, treatment with a PPAR-α antagonist significantly attenuated social impairment, repetitive behavior, hyperactivity, anxiety, and low exploratory activity in environmental models of ASD [257, 258]. Thus, we speculate that the therapeutic effects of PEA can be potentiated through the combination of PEA and modulators of GPR55, PPAR-γ, and PPAR-α activity.

Modulation of PPAR-α and TRPV1 also shows therapeutic potential for treating GI disorders such as gut dysbiosis, permeability issues, and systemic inflammation, which are often comorbid in ASD. This modulation can be achieved through the respective ligands of these receptors, such as PEA, *N*-oleoylethanolamine, as well as the TRPV1 agonist capsaicin [54, 259–261]. Interestingly, in FXS, the concentrations of eCBs was not altered; however, the receptors and enzymes involved in the metabolism of eCBs, such as FAAH, MAGL, CB1 on GABAergic terminals, and mGlu5R, were affected [246, 247].

In conditions like ASD and FXS, increased levels of mucin-degrading bacteria (e.g., Verrucomicrobiota and *Akkermansia*) can serve as biomarkers for gut barrier integrity, permeability, and inflammation [33, 36, 37, 39]. Higher levels of these bacteria may impair hippocampal function and cognitive processes related to neuronal development [262, 263], potentially exacerbating psychiatric symptoms through a disrupted gut–brain axis [38]. Interestingly, while the species *A. muciniphila* has been associated with beneficial effects in restoring gut barrier function and reducing intestinal permeability, it also possesses inflammatory properties due to increased exposure of immune cells to microbial antigens when the mucosal layer is compromised [36]. This double-edged role may contribute to inflammatory conditions, as suggested not only in ASD but also in neurodegenerative diseases like multiple sclerosis and Parkinson’s disease [36]. Nevertheless, in a mouse model of metabolic syndrome, supplementation with *A. muciniphila* improved gut permeability and increased the levels of 2-AG and its mono-acyl-glycerol congeners [264]. In obese and overweight men, supplementation of this bacterium elevated the circulating levels of eCBome mediators, 1-palmitoyl-glycerol (1-PG) and 2-palmitoyl-glycerol (2-PG), which bind to PPAR-α receptors with beneficial effects on lipid metabolism and energy regulation [265]. Positive modulation of PPAR-α and TRPV1 by eCBome-active GM compounds could thus offer therapeutic benefits in treating ASD and associated GI and metabolic symptoms.

Additionally, fecal microbial transplantation from wild-type mice into *Fmr1*^*−/y*^ mice increased *A. muciniphila* levels, while simultaneously ameliorating cognitive and social deficits [266]. This raises uncertainty about whether *A. muciniphila* is strictly beneficial or detrimental in the context of the *Fmr1* mutation. At any rate, the mucin-degrading activity of *A. muciniphila* might not necessarily imply a negative role in neurodegenerative and neuropsychiatric disorders [36], as such activity might become pathological only after that other more pathogenic bacteria have already compromised the mucosal intestinal barrier, as suggested by the fact that the relative abundance of this species is decreased in obesity [267].

Therefore, *Verrucomicrobiota*, and specifically *A. muciniphila*, can modulate eCBome mediator levels. This makes them both potential biomarkers and therapeutic targets for ASD-related symptoms and GI pathophysiology.

In the valproic acid-exposed and BTBR models of ASD, treatment with butyrate improved recognition memory and social behavior [166, 167]. Butyrate modulates eCB tone in gut epithelial cells by regulating enzymes involved in eCBs synthesis and breakdown, such as *N*-acyl-phosphatidylethanolamine-specific phospholipase D (NAPE-PLD), DAGL-α, MAGL [268] and FAAH [269]. This modulation indicates that excessive butyrate levels may alter eCBs levels in ASD. However, clinical studies have reported conflicting findings regarding the abundance of butyrate-producing bacteria and fecal butyrate levels in ASD populations [270–272], mostly concerning *Faecalibacteria* and *Faecalibacterium prausnitzii* concentrations. Further research is needed to determine whether modulating butyrate-producing bacteria is beneficial or detrimental for addressing the dysregulated eCBome–GM–brain axis in ASD.

Endogenous cannabinoids are substrates for cyclooxygenase-2 (COX-2) and can be oxygenated by COX-2 to form new classes of prostaglandins [273]. The signaling of two eCBome mediators, prostaglandin E2 (PGE2) and COX-2, is altered in ASD [274]. As PGE2 plays a crucial role in brain development, abnormalities in the COX-2/PGE2 pathway have been linked to ASD [274, 275]. Studies report lower arachidonic acid levels alongside increased COX-2 and PGE2 in the blood plasma of individuals with ASD [276, 277], correlating these changes with sensory abnormalities [277]. Polymorphisms in the *PTGS2* gene, which encodes COX-2, have also been associated with hyperactivity and atypical communication in ASD [278]. Misuse of misoprostol (a PGE2 analogue) during early pregnancy is linked to a higher incidence of neurodevelopmental disorders, including Moebius syndrome and ASD [279–281]. Moreover, COX-1/2 deficient mice display ASD-like behaviors, such as hyperactivity, repetitive behaviors, and atypical social interaction [274], alongside disruptions in ASD-related biological pathways [275]. Therefore, the COX-2/PGE2 pathway also represents a viable potential therapeutic target in ASD.

### The eCBome–GM–brain axis in ASD, irritability and aggressivity

The challenges faced by individuals with ASD in articulating their own mental and emotional states, recognizing those of others, and coping with sensory sensitivity, unmet needs, and societal criticism often lead to discrimination and social isolation [282], as well as increased irritability, meltdowns, tantrums, or self-injurious behaviors [283].

In preclinical models, social isolation disrupts brain development, particularly in the ventral hippocampus (vHPC), increasing excitatory activity and exacerbating stress-induced impulsive aggression [217]. Activation of the CB1 receptor by agonists like arachidonylcyclopropylamide reduces aggression in stress tests without affecting overall activity [217]. CB1 activation also elevates levels of eCBs such as AEA and 2-AG in the vHPC, and by inhibiting c-Fos expression, mitigates biting, offensive behavior, and impulsive aggression in rodents [217–219]. Additionally, in preclinical models, enhancing 2-AG signaling through MAGL inhibition reduced aggressiveness while increasing victimization [284], and cannabidiol decreased social isolation-induced aggressive behaviors likely by activating both CB1 (indirectly) and 5-HT1 A receptors [285]. Interestingly, also acute administration of the CB2 agonist significantly reduced isolation-induced aggressivity [286], while CB2 receptor downregulation showed increased aggressivity [286, 287]. The findings suggest that disruptions in social interaction and isolation contribute to heightened aggression and altered brain activity, particularly in the vHPC, and that targeting the endocannabinoid eCBome—via CB1 and CB2 receptor activation or MAGL inhibition—may mitigate these effects, highlighting potential therapeutic strategies for managing aggression and social deficits in ASD.

*Clostridium*, a bacterium whose abundance is negatively associated with CB1 and CB2 activity [288], correlates positively with irritability in ASD symptoms [289, 290]. At the neuronal level, the gut abundance of *Clostridium*, and specifically the taxa *Coprococcus*, shows negative associations with changes in critical analysis and reasoning skills in children with ASD [115]. Therefore, CB1 activation can potentially suppress impulsive aggression by increasing AEA and 2-AG [217–219] and reducing *Clostridium* abundance [288]. The eCB system appears to regulate both neuronal activity and GM composition related to irritability and aggression, suggesting it could be a therapeutic target for individuals with ASD experiencing aggressive or self-injurious behaviors.

While current treatments like probiotics for ASD often include *Streptococcus* strains [291], these taxa are found to be significantly increased in children with ASD and irritability. Another overabundant bacterium in ASD-related irritability is *Alistipes* [289], a SCFA-producing bacterium primarily generating propionate and acetate, which may contribute to neurotoxicity and dysbiosis [225]. Notably, acetate administration is used to model ASD epigenetically due to its neurotoxic effects. Furthermore, propionic acidemia induces ASD-like behaviors, possibly by altering neurotransmitters such as dopamine [227, 292, 293]. Inhibition of CB1 receptors has been shown to increase levels of both propionate-producing bacteria and dopamine [231]. Hence, targeting the negative interaction between CB1 and *Alistipes* may hold promise as a therapeutic approach for managing ASD-related aggressivity and related comorbid behaviors.

### The eCBome–GM–brain axis in ASD, ADHD and OCD

The role of the eCBome as a therapeutic target in ADHD remains unclear. ADHD is associated with dysregulated dopamine signaling [294], which may affect eCB levels [295]. Increased AEA levels have been observed in ADHD, possibly due to hyperactive dopamine D2 receptors [295]. Preclinical models of ADHD display a loss of striatal CB1 receptors on GABAergic terminals and a dysfunctional FAAH polymorphism [296, 297]. Interestingly, MAGL and FAAH inhibition in rats induced ADHD-like behaviors, which were reversed by a CB1 receptor inverse agonist [298]. Conversely, MAGL inhibition in models with hyperactivity, such as *Fmr1* mice, restored eCB-mediated synaptic plasticity, and reduced hyperactivity, anxiety, and other ASD behaviors [47, 246, 247]. Additionally, CB1 antagonists improved memory impairments in the *Fmr1* model of ASD [49].

Also, treatment with a PPAR-α antagonist significantly attenuated hyperactivity, anxiety, and other ASD behaviors in models of ASD [257, 258].

Short chain fatty acids production alterations are implicated in ADHD. Among SCFAs, butyrate modulates the expression of eCBome enzymes such as MAGL, NAPE-PLD and DAGL in a dose-dependent manner [268], as well as modulating neurotransmitter (e.g., GABA) levels [166] and CB1 receptors signaling [299].

In ASD with ADHD, there is increased abundance of *Odoribacter*, particularly *O. splanchnicus*, whereas species from the Ruminococcaceae family, known for butyrate production, like *F. prausnitzii*, are decreased. *F. prausnitzii* has been negatively associated with ADHD severity and hyperactivity [300]. However, *Ruminococcacae_UGC* was positively correlated with the risk of ADHD and with lack of attention [301, 302], potentially due to its association with microbial species capable of affecting GABA neurotransmitter levels [303]. We therefore speculate that alterations in SCFA levels might affect the availability of the eCBs AEA and 2-AG, impacting synaptic plasticity and neurotransmitters implicated in ADHD pathophysiology.

Variation of a genus from the Lachnospiraceae family (*Coprococcus*) showed a trend of being negatively associated with inattention symptoms [301, 302]. The genera *Butyricicoccus*, *Roseburia*, *Desulfovibrio*, *Lachnospiraceae NC2004 group*, and *Romboutsia* were protective factors for ADHD, with the abundance of the genus *Butyricicoccus* being negatively correlated with the risk of ADHD [302]. The differential abundance of gut bacteria in ADHD, as reviewed elsewhere [303], shows distinct patterns from that observed in ASD. This suggests unique microbial signatures associated with either condition. Together, further research is needed to uncover the interplay between SCFA-producing bacteria and the eCBome in ADHD comorbid with ASD.

Obsessive–compulsive disorder involves complex neurobiological mechanisms, including altered brain-derived neurotrophic factor (BDNF) expression and oxytocin signaling [304, 305], both of which interact with the eCBome [204, 306–309]. In a translational study on blood samples from individuals with OCD and OCD rat models, downregulation of enzymes responsible for eCB biosynthesis, such as DAGLα and NAPE-PLD, was correlated with reduced oxytocin receptor expression. Moreover, CB1 and CB2 receptor expression was downregulated, with a significant inverse correlation observed between BDNF and CB2 gene expression [309]. These findings underscore intricate relationships between the eCBome, BDNF, and oxytocin signaling in OCD and its co-occurrence with ASD.

Indeed, as mentioned above, many eCBome components have beneficial effects in ASD co-occurring with OCD behavior. Treatment with a PPAR-α antagonist and PEA significantly attenuated repetitive [54, 250, 257, 258] and stereotyped behaviors in ASD models [54, 250], while COX-1/2 deficient mice display repetitive behaviors alongside ASD-like behaviors [274, 275].

Differences in GM taxonomic composition, particularly with regard to SCFA-producing bacteria, such as Lachnospiraceae and Ruminococcaceae, have been noted in OCD models and ASD [310, 311]. Specifically, genera such as *Anaerostipes* and *Odoribacter* were reduced in individuals with OCD, while in the *Fmr1* mouse model of ASD they were over-abundant [33, 310]. The genus *Oscillospira* instead was under-represented in OCD models, mirroring observations in the *Fmr1* knockout mouse model [310, 312].

In a preclinical model of OCD, a lower abundance of *Prevotella* and *Anaeroplasma*, both known for their anti-inflammatory properties, was observed [313], consistent with gut microbiota composition findings in ASD [314]. Youth suffering from pediatric autoimmune neuropsychiatric disorders associated with streptococcal infections (PANDAS), which exhibit OCD-like symptoms [315], showed a decrease in the genera *Roseburia* and *Coprococcus*, but an increase of *Odoribacter* and *Oscillospira* [316], contrasting with previous studies in OCD individuals [310]. The genera *Roseburia* and *Coprococcus* are known for their protective properties for gut health and are also reduced in ASD and FXS. Kamble and Dandekar reviewed a wide spectrum of bacteria involved in OCD, considering its comorbidity with other psychiatric disorders, although they did not discuss specific links to ASD [316].

## Conclusions

The eCBome–GM–brain axis is emerging as a promising therapeutic target for ASD and its systemic and psychiatric comorbidities. Dysregulated eCB levels, particularly reduced OEA and AEA concentrations and altered CB1 receptor activity, may contribute to ASD symptoms. Modulating this axis, through inhibitors of FAAH, TRPV1, and MAGL holds potential for sociability impairments, depression and anxiety. However, inhibition of FAAH and MAGL may induce ADHD-like behaviors, which could be reversed with a CB1 receptor inverse agonist. Conversely, MAGL inhibition may restore eCB-mediated synaptic plasticity, reduce hyperactivity, anxiety, aggressivity and improve repetitive, stereotypical, and sensory behaviors. Targeting mGlu5R to elevate 2-AG through DAGL activation could also provide therapeutic benefits in ASD, while the COX-2/PGE2 pathway may serve as another target to manage hyperactivity and repetitive behaviors. Supplementation with PEA has shown promise in improving repetitive and stereotyped behaviors, language skills, and GI symptoms, including intestinal permeability and inflammation. GPR55, PPAR-γ, and PPAR-α receptors may hold further therapeutic promise within this axis, particularly through modulation by PEA. Targeting CB1 and CB2 receptor activation may mitigate aggressivity in ASD.

Mucin-degrading bacterial taxa represent potential targets for managing ASD symptoms and GI pathophysiology. Selective antibiotics against specific *Clostridium* strains may improve irritability and aggression. Changes in SCFAs levels, for example through prebiotics, may influence eCB availability, impacting synaptic plasticity and neurotransmitter systems. Anti-inflammatory bacteria like *Prevotella* and *Anaeroplasma*, alongside gut health biomarkers such as *Roseburia* and *Coprococcus*, may offer therapeutic value. Conflicting findings regarding butyrate-producing bacteria in ASD populations suggest caution when using these bacteria as targets. Further research is needed to elucidate their role in modulating the eCBome–GM–brain axis in ASD.

In conclusion, the eCBome–GM–brain axis represents a promising, multifaceted therapeutic target for ASD and its comorbidities, warranting further clinical and preclinical research to clarify its therapeutic potential and refine targeted interventions.

## Supplementary Information

Additional file 1.

## Data Availability

No data and material were used for the research described in the article.

## Abbreviations

1-PG: 1-Palmitoylglycerol
2-AG: 2-Arachidonoylglycerol
2-PG: 2-Palmitoylglycerol
AEA: Anandamide
ADHD: Attention deficit hyperactivity disorder
ASD: Autism spectrum disorder
BBB: Blood–brain barrier
CB1: Cannabinoid receptor type 1
c-Fos: Cellular proto-oncogene Fos
CNVs: Copy number variations
DAGLα: Diacylglycerol lipase alpha
eCB: Endocannabinoid
eCBome: Endocannabinoidome
FAAH: Fatty acid amide hydrolase
FMR1: Fragile X messenger ribonucleoprotein 1
FXS: Fragile X syndrome
GABA: Gamma-aminobutyric acid
GI: Gastrointestinal
GM: Gut microbiome
GPR55: G-Protein-coupled receptor 55
GPR119: Oleoyl-lysophosphatidylinositol receptor 1
MAGL: Monoacylglycerol LIPASE
MECP2: Methyl-CpG binding protein 2
mGlu5R: Metabotropic glutamate receptor 5
NAE: *N*-Acylethanolamines
NAPE-PLD: *N*-Acyl phosphatidylethanolamine phospholipase D
NF1: Neurofibromatosis type 1
OCD: Obsessive-compulsive disorder
OEA: Oleoylethanolamide
PEA: Palmitoylethanolamide
PMS: Phelan–McDermid syndrome
PPAR-α: Peroxisome proliferator-activated receptor alpha
PPAR-γ: Peroxisome proliferator-activated receptor gamma
RTT: Rett syndrome
SHANK3: SH3 and multiple ankyrin repeat domains 3
TRPV1: Transient receptor potential vanilloid 1
TSC: Tuberous sclerosis complex (1 and 2)
vHip: Ventral hippocampus
VMH: Ventromedial hypothalamus
VPA: Valproic acid
