# Supplementary material for: The endocannabinoidome–gut microbiome–brain axis as a novel therapeutic target for autism spectrum disorder
Source: J Biomed Sci. 2025 Jul 2;32:60. doi: 10.1186/s12929-025-01145-7 (PMC12220735; doi:10.1186/s12929-025-01145-7)
Supplement: Supplementary file 1 — Additional file 1. [file 12929_2025_1145_MOESM1_ESM.docx]

| **Category / approach / design** | **Participants** | **Treatment Duration** | **Outcomes** | **Clinicaltrial.gov**  **Identifier / status** | **Title** |
| --- | --- | --- | --- | --- | --- |
| Cannabis-based therapies  /  CBD-rich cannabis oil (CBD:THC ratio of 20:1)  /  Open-label, Randomized,  parallel assignment | 5-25 years old children/adults with ASD | Titration till improvement in some parameters is seen or till treatment failure decision is made | PRIMARY  -Cannabinoids levels  -Attention span  -Cognition  -Adaptive behavior  -Violent behavior  SECONDARY  -Side effects  -Therapeutic failure  -Sleep problems  -Eating problems  -Hormonal profiles  -Biochemical parameters | NCT05212493  /  Completed | The Effects of Medical Cannabis in Children with Autistic Spectrum Disorder |
| Cannabis-based therapies  /  CBD-rich cannabis flowers dissolved in olive oil (30% CBD and 1.5% Δ9-THC)  /  Double-blind, randomized, placebo-controlled crossover | 2-8 years old children with ASD | 12 weeks per phase (crossover study) | PRIMARY  -ASD symptoms  SECONDARY  -Clinical global impression  -Sensory profile score  -Eye movements  -Sleep architecture | NCT05413187  /  Withdrawn due to COVID-19 | A Trial to Assess the Efficacy and Safety of Medical Grade Cannabis in Children Diagnosed with Autism Spectrum Disorder |
| Cannabis-based therapies  /  Oil-based tincture (23:1 ratio of CBD to Δ9-THC)  /  Double-blind, randomized, placebo-controlled, parallel assignment | 18-45 years old adults with ASD | 8 weeks.  -Start at 50 mg of CBD and 2.18 mg of THC per day.  -Increase to 200 mg of CBD, and 8.7 mg of TCH by week 4.  -Weeks 5 through 8 will be at the maximum dose 400 mg of CBD and 17.4 mg of THC. | PRIMARY  -anxiety symptoms  SECONDARY  -Socialization  -Repetitive behaviors  -Aberrant behaviors | NCT06526208  /  Not yet recruiting | Investigating a Marijuana-based Compound as a Treatment for Anxiety in Autistic Adults |
| Cannabis-based therapies  /  NTI164 (full-spectrum medicinal cannabis plant extract with less than 0.08% THC)  /  Double-blind, parallel assignment, randomized and controlled-to-open-label | 8-17 years old children with ASD | 18 to 54 weeks.  Baseline dose of 5 mg/kg/day that will be increased weekly by 5 mg/kg for a period of 4 weeks until the maximum tolerated dose or 20 mg/kg/day is achieved. | PRIMARY:  -Clinical global impression severity  SECONDARY:  -Adaptive behavior  -Social responsiveness  -Anxiety, depression, and mood  -Sleep disturbances  -Caregiver impression of attention  -Caregiver impression of target behavior | NCT05626959  /  Unknown status | Evaluating the Efficacy of NTI164 in Young People with Autism Spectrum Disorder |
| Cannabis-based therapies  /  FEN164 (full-spectrum medicinal cannabis plant extract -oil based- with less than 0.08% THC)  /  Open label, single group | 8-17 years old children with ASD | 20-week.  Stage 1: 5mg/kg, 10mg/kg, 15mg/kg, 20mg/kg (1 week each) Stage 2: 20mg/kg (8 weeks), 15mg/kg, 10mg/kg, 5mg/kg (1 week each) | PRIMARY:  -Clinical global impression improvement  SECONDARY:  -Adaptive behavior  -Social responsiveness  -Anxiety, depression, and mood  -Sleep disturbances  -Autism family experience  -Caregiver impression of attention  -Caregiver impression of target behavior | NCT05516407  /  Unknown status | Safety and Efficacy of Oral Full-Spectrum Medicinal Cannabis Plant Extract in Children with Autism Spectrum Disorder. |
| Cannabis-based therapies  /  CBD softgel capsules  /  Double-blind, randomized, crossover | Over 18 years old adults with ASD | 6 weeks.  CBD, starting at 100 mg twice daily, and increased to 200 mg twice daily by week 3. | PRIMARY:  -Aberrant behavior  SECONDARY:  -Anxiety  -OCD symptoms  -Clinical global improvement  -Neuropsychiatric symptoms  -Daily living  -Social communication  -Mental state  -Adverse effects | NCT05015439  /  Recruiting | Cannabidiol (CBD) in Adults With ASD |
| Cannabis-based therapies  /  single acute dose of CBD or cannabidivarin  /  Double-blind,  randomized, crossover | 18 to 50 years old  Male adults with ASD  neurotypical male adults | Single acute oral dose of CBD or cannabidivarin, 600 mg | PRIMARY:  -Balance of excitatory and inhibitory neurotransmitters ([1H]MRS).  SECONDARY:  -fMRI low-frequency bran activity  -fMRI resting state functional connectivity | NCT03537950  /  Completed | Shifting Brain Excitation-Inhibition Balance in Autism Spectrum Disorder |
| Cannabis-based therapies  /  Epidiolex Oral Solution (FDA-approved purified CBD product)  /  Double-blind, randomized, crossover | 7-14 years old | 8 weeks.  Week 1: 5 mg/kg/day, divided into 2 doses Week 2: 10 mg/kg/day, divided into 2 doses Weeks 3-8: 20 mg/kg/day, divided into 2 doses | PRIMARY:  -Repetitive behavior  -Child behavior checklist  -ADOS-2  SECONDARY:  -Aberrant behavior  -Self-harm  -Social responsiveness  -Vocabulary test  -Sleep habits  -MRS to measure neurotransmitters levels | NCT04517799  /  Active, not recruiting | Trial of Cannabidiol to Treat Severe Behavior Problems in Children with Autism |
| Cannabis-based therapies  /  Epidiolex Oral Solution (FDA-approved purified CBD product)  /  Double-blind, randomized, placebo-controlled, crossover | 6-17 years old | 12 weeks.  5 milligrams per kilogram per day (mg/kg/day) GWP42003-P for 1 week and then 10 mg/kg/day GWP42003-P for 11 weeks. | PRIMARY:  -Aberrant behavior  -Adaptive behavior  -Clinical global impression  SECONDARY:  -Severe treatment-emergent adverse events  -Abnormal laboratory parameter values  -Abnormal physical examination  -Abnormal ECG  -Suicidal behavior | NCT04745026  /  Completed | Trial to Investigate the Safety and Efficacy of Cannabidiol Oral Solution (GWP42003-P; CBD-OS) in Children and Adolescents with Autism Spectrum Disorder |
| Cannabis-based therapies  /  Epidiolex Oral Solution (FDA-approved purified CBD product)  /  Double-blind, randomized, placebo-controlled, crossover, | 5-17 years old | 12 weeks.  Titration dose 5 mg/kg/day (1^st^ and 12^th^ week). Treatment dose 10 mg/kg/day (2^nd^-11^th^ week). | PRIMARY:  -Aberrant behavior  SECONDARY:  -Lethargy/social withdrawal  -Stereotypic behavior  -Inappropriate speech  -Hyperactivity/ noncompliance  -Anxiety, depression, and mood  -Avoidance  -Social responsiveness  -Autism severity  -Interaction assessment  -Repetitive behavior  -Executive functioning  -ADHD  -Sleep quality  -Family experience  -Social role and activities  -Life satisfaction  -Positive affect  -Quality of life | NCT04520685  /  Active, not recruiting | CASCADE: CAnnabidiol Study in Children with Autism Spectrum DisordEr (CASCADE) |
| Cannabis-based therapies  /  98% pure CBD  /  Open label, sequential assignment | 7-17 years old  Male and female children with ASD | 6 weeks.  9 mg/kg/day | PRIMARY:  -Global clinical impression  SECONDARY:  -Repetitive behavior  -Social Responsiveness  -Aberrant behavior  -Anxiety  -Sleep disturbance  -Adaptive behavior  -Autism family experience  -Behavioral inflexibility  -ADHD | NCT03900923  /  Completed | Cannabidiol for ASD Open Trial |
| Cannabis-based therapies  /  -Pure CBD and Δ9-THC (20:1 ratio)  - Whole plant extract enriched with CBD and THC (20:1 ratio)  /  Randomized, double-blind, placebo-controlled, parallel assignment | 5-21 years old  Male and female children with ASD | 12 weeks.  Starting at 1 mg/kg CBD per day, up-titrated until intolerance or to a maximum dose of 10 mg/kg CBD per day, divided to 3 daily doses. | PRIMARY:  -ASD symptom severity  -Clinical global impression  SECONDARY:  -Social responsiveness  -Parenting stress  -Adverse events | NCT02956226  /  Completed | Cannabinoids for Behavioral Problems in Children with ASD (CBA) |
| Cannabis + endocannabinoids based therapies  /  CBD oil combined with PEA (Palmitoylethanolamide)  /  Double-blind, randomized, controlled, crossover | 5-18 years old  Male and female children with ASD | 24 weeks.  Oral CBD oil plus pills of CannAmide (palmitoylethanolamide (PEA) 400 mg twice daily | PRIMARY:  -Safety  -Clinical global impression  -Aberrant behavior  SECONDARY:  -Safety  -Tolerability  -Social responsiveness  -Sleep  -ASD symptoms  -Adaptive behavior  -Language abilities  -Eye-tracking | NCT05182697  /  Not yet recruiting | SCI-210 in the Treatment of Children and Young Adults with AutismEvaluate the Safety, Tolerability and Efficacy of SCI-210 in Children with Autism Spectrum Disorder (ASD) |
| Endocannabinoids based therapies  /  PEA (Palmitoylethanolamide)  /  Open label, single group | 18-35 years old  adults with ASD | 12-24 weeks.  Oral ultramicronized PEA 600 mg/day.  During the 24-week extension 600-1200 mg/day) | PRIMARY:  -Global symptoms  SECONDARY:  -Disability  -Anxiety  -Depression  -Somatization | NCT06187090  /  Recruiting | The Supplementation Therapy in Autism and Response to Treatment Study (START) |

**Supplementary table 4. Clinical trials of cannabis-based therapies in ASD**
